# Inappropriateness of Antibiotic Prescribing in Medical, Surgical and Intensive Care Units: Results of a Multicentre Observational Study

**DOI:** 10.3390/life11060475

**Published:** 2021-05-24

**Authors:** Margherita Macera, Federica Calò, Lorenzo Onorato, Giovanni Di Caprio, Caterina Monari, Antonio Russo, Anna Galdieri, Antonio Giordano, Patrizia Cuccaro, Nicola Coppola

**Affiliations:** 1 Department of Mental Health and Public Medicine. University of Campania Luigi Vanvitelli, 81100 Caserta, Italy; macera.margherita@policliniconapoli.it (M.M.); federica.calo@policliniconapoli.it (F.C.); lorenzoonorato@unicampania.it (L.O.); caterina.monari@policliniconapoli.it (C.M.); antonio.russo@studenti.unicampania.it (A.R.); 2 Infectious Diseases Unit, AORN Sant’Anna and San Sebastiano, Caserta Hospital, 81100 Caserta, Italy; giovanni.dicaprio@ospedale.caserta.it; 3 Direzione Sanitaria, AOU Vanvitell, University of Campania, 80138 Naples, Italy; anna.galdieri@policliniconapoli.it; 4 Direzione Generale, AOU Vanvitell, University of Campania, 80138 Naples, Italy; antonio.giordano@policliniconapoli.it; 5 Direzione Sanitaria, AORN Sant’Anna and San Sebastiano, Caserta Hospital, 81100 Caserta, Italy; patrizia.cuccaro@ospedale.caserta.it

**Keywords:** antimicrobial resistance, antibiotic prescription, antibiotic appropriateness

## Abstract

The objectives of the present study were to provide a snapshot analysis of antibiotic appropriateness in two hospitals in Southern Italy in three specific areas, surgical, medical and intensive care, and to evaluate the risk factors associated with inappropriateness in antimicrobial prescriptions. We conducted a multicentre observational study in two hospitals in the Campania region. We collected data of all patients admitted on the day of evaluation to antibiotic therapy or prophylaxis through a case report form. The primary outcome was to assess the inappropriateness of antibiotic prescribing, related to the spectrum, dose, route of administration and duration of treatment—in particular, to assess whether there was a difference in the adequacy of the prescriptive practice in the medical, surgical and intensive sectors. Prescriptive inappropriateness was more frequently observed in surgical units (79.8% of the 104 antimicrobial prescriptions) than in medical units (53.8% of the 65 prescriptions, *p* = 0.0003) or in intensive care units (64.1% of the 39 prescriptions, *p* = 0.052). The reasons for the inappropriate antimicrobial prescriptions were similar in the three areas evaluated: antimicrobial unnecessary and antimicrobial not recommended were the most frequent reasons for inappropriateness. Not participating in an antimicrobial stewardship program (ASP) was identified as a factor associated with inappropriate antimicrobial prescriptions in medical and surgical units, but not in Intensive Care Units (ICUs). ASPs may enhance the appropriateness of antimicrobial prescriptions especially in medical and surgical units. In ICUs, specific programs able to limit empirical therapies and encourage the collection of microbiological samples may be useful to set up targeted therapies and to design antimicrobial protocols.

## 1. Introduction

Antibiotic resistance is a major public health problem and antimicrobial overuse is considered the principal driver in selecting for antimicrobial-resistant organisms, with a strong association between the rising rates of use and increasing rates of antimicrobial-resistant organisms [1,2]. Therefore, it is important to reduce the inappropriate use of antibiotic therapy [3]. In fact, not all antimicrobial prescriptions are indicated, and when the use of antibiotics is required, the spectrum, dose, route of administration and duration of treatment should be optimised [4,5,6]. It is estimated that 30 to 50% of antibiotics are prescribed inappropriately, as defined by low adherence rates to antibiotic prescription guidelines [7]. We should consider, however, that the definition of inappropriateness could vary according to the setting and is not always clear-cut. [8]

Improving antibiotic prescriptions in the hospital setting is of the utmost importance to preserve their effectiveness. Moreover, knowing the degree of inappropriateness in antimicrobial prescriptions and the factors associated may be useful in the design of strategies to reduce the development of multidrug-resistant microorganisms.

The objectives of the present study were to provide a snapshot analysis of antibiotic appropriateness in two hospitals in Southern Italy in three specific areas, surgical, medical and intensive care, including both adult and paediatric patients, and to evaluate the risk factors associated with inappropriateness in antimicrobial prescriptions, such as the absence of an antimicrobial stewardship program in the unit. We chose to group the wards according to the area of interest (medical, surgical or critical area) because the prescribing behaviour can significantly vary among physicians of different specialties. For example, an ethnographic study published a few years ago had demonstrated that, in medical wards, the antimicrobial prescribing activity was often considered the result of a collective decision-making process, involving different specialties, while in surgical units, it is more individualised and often relegated to junior physicians [9].

## 2. Patients and Methods

This study was conducted in two hospitals in Naples and Caserta, in Southern Italy. The medical institution located in Naples includes 30 units for a total of 268 beds and does not have an emergency department; the community hospital in Caserta has 23 wards accounting for 486 active beds with an emergency department. All units of the two hospitals were included in the analysis; the list of wards, grouped according to the clinical area, is displayed in Supplementary Tables S1 and S2. A team of infectious disease consultants launched a persuasive educational antimicrobial stewardship program (ASP), based on audit and feedback in some departments in both hospitals, in January 2017 in Naples and in April 2018 in Caserta. The ASP team devised diagnostic and therapeutic protocols based on national and international guidelines on the main infectious syndromes. However, these protocols have been shared with all units, whether participating or not in ASP.

We conducted a prevalence study that was planned in accordance with the health directions of the two hospitals in order to analyse the antibiotic use and to evaluate the appropriateness of antibiotic prescribing; the assessment was performed through audits conducted in each facility, on 10–14 of February 2020 in Naples and 14–23 of November 2019 in Caserta. The infectious disease (ID) consultants involved received the same training on local and international guidelines on the main infectious syndromes and prepared a pre-formed case report form. The audits in each hospital were managed by ID consultants operating in the other centre.

During the audits, the following data were collected: type of unit (i.e., medical, surgical or ICU), number of patients admitted to the ward, number of patients receiving an empiric or targeted antimicrobial treatment and/or prophylaxis (agent prescribed, dosage, route of administration, indication and duration). For each antibiotic prescription, the team of ID specialists assessed the appropriateness in relation to the patient’s characteristics and the indication provided by national and international guidelines. All classes of antimicrobials for systemic use were included in the analysis. Topical antibiotics were excluded because they have a limited impact on the selection of resistance and were mostly unavailable in the formulary of both hospitals.

For each patient undergoing antibiotic therapy, we collected the following data: type of acquisition of infection (nosocomial according to US Center for Disase Control (CDC) criteria [10], healthcare-associated or community-acquired), disease severity (i.e., diagnosis of sepsis or septic shock [11], the site of infection and whether the therapy was empirical or targeted.

### 2.1. Definitions

A therapy was defined as empirical when administered before the identification of the causative agent with the relative resistance profile, or targeted otherwise. The appropriateness of all prescriptions was evaluated in accordance with the international guidelines on the different clinical syndromes [12,13,14,15,16]. Considering that different guidelines can vary in their recommendations, we decided to use those based on the most recent available evidence and those best suiting the local epidemiology of resistance, particularly for empirical therapy. Targeted treatment was defined as appropriate if it displayed in vitro activity against the clinical isolate and if it was correct in dosage, duration and the method of administration. Empirical therapy was considered appropriate when it included at least one antimicrobial with activity against the most frequent pathogens involved in the clinical syndrome presented by the patient.

For every antibiotic prescription considered inappropriate, the reason for inappropriateness was recorded; more than one reason could be reported for each prescription. The antibiotic was considered unnecessary when the patient’s clinical conditions did not show evidence of infection requiring an antimicrobial treatment. The therapy was considered inadequate or inactive when not able to cover the most likely causative agents, as indicated by the current guidelines (for empirical treatment), or when the agent displayed no activity against the pathogen involved (for targeted treatment).

Antimicrobial therapy was considered not recommended when the antibiotic was active against the identified or presumed aetiologies but showed an excessively wide spectrum of activity. The duration of antimicrobial therapy was considered excessive when it exceeded the limit indicated by the international guidelines.

### 2.2. Outcomes

The primary outcome was to assess the difference in the appropriateness of prescriptive practice in the medical, surgical and intensive care units.

The secondary outcome was to identify the main drivers of prescriptive inappropriateness in order to implement strategies to optimise antibiotic prescribing in the three areas.

### 2.3. Ethics

All methods used in the study were in accordance with the international guidelines, with the standards on human experimentation of the Ethics Committee of the Azienda Ospedaliera Universitaria, University of Campania and with the Helsinki Declaration of 1975 and revised in 1983. Since the leadership of the University of Campania and Azienda Ospedaliera of Caserta formally approved the study and the data were collected during routine clinical activities, no approval was required by the local ethics committee.

### 2.4. Statistical Analysis

Continuous variables were summarised as mean and standard deviation, and categorical variables as absolute and relative frequencies. For continuous variables, the differences were evaluated by the Student t-test; categorical variables were compared by the chi-square test, using Fisher’s exact test when needed; odds ratios (OR) and their 95% confidence intervals were calculated for each comparison, as well as the chi-square test values. The sample size needed to obtain a statistical power of 80%, with a probability of type I error of 0.05 and an anticipated difference in the prevalence of inappropriate prescriptions of 20% between medical and surgical units and of 25% between surgical units and ICUs, was calculated.

## 3. Results

### 3.1. Characteristics of Units Participating in the Study

In the two hospitals, a total of 53 units were assessed; in particular, 19 medical, 25 surgical and 9 intensive care units (ICUs) were evaluated. The total number of patients assessed was 486: 210 in medical, 187 in surgical and 89 in ICUs (Supplementary Table S3). Of the 210 patients in medical units, 65 (31%) were being treated with antibiotics (6.2% in antibiotic prophylaxis and 93.8% in antibiotic therapy), 104 (55.7%) of the 187 patients in surgical units (66.3% in antibiotic prophylaxis and 33.7% in antibiotic therapy) and 39 (43%) of the 89 patients in ICUs (12.8% in antibiotic prophylaxis and 87.2% in antibiotic therapy).

In Supplementary Table S4, the prescriptions of the different antimicrobials are shown according to the medical, surgery and ICUs.

### 3.2. Inappropriateness in Antimicrobial Prescription

In Table 1, we present the characteristics of antimicrobial prescriptions stratified according to their appropriateness; ORs with 95% Confidence Intervals (CI) and chi square values are shown for each comparison. Prescriptive inappropriateness was more frequently observed in surgical units (79.8% of the 104 antimicrobial prescriptions) than in medical units (53.8% of the 65 prescriptions, OR 3.39, 95% CI 1.71–6.71, *p* = 0.0003) and ICUs (64.1% of the 39 prescriptions, OR 2.21 95% CI 0.98–4.98, *p* = 0.052) (Table 1).

As shown in Table 1, the reasons for inappropriate antimicrobial prescription were similar in the three areas evaluated, and antimicrobial unnecessary and antimicrobial not recommended were the most frequent reasons for inappropriateness (Table 1). However, an unnecessary antibiotic was more frequently (48%) recorded in ICUs, while an inadequate method of administration (14.3%) was recorded in medical units (Table 1). Not infrequently (20–27.7%), two reasons for inappropriate prescribing were observed (Table 1).

### 3.3. Factors Associated with Inappropriateness in Medical Units

Table 2 shows the factors associated with inappropriateness in antimicrobial prescriptions (Table 2). No difference was observed in the acquisition of infection, severity and source of infection between appropriate and inappropriate antimicrobial prescriptions (Table 2). However, inappropriate antimicrobial prescriptions were more frequently identified in units not participating in an ASP (80.7% vs. 46.7%, OR 9.33, 95% CI 2.34–37.2, *p* = 0.0006), and less frequently in the case of endocarditis or cardiovascular infections (0% vs. 16.7%, *p* = 0.02) and when microbiological specimens (hemocultures) were collected (58.1% vs. 83.3%, *p* = 0.048) (Table 2). Multivariable analysis of the factors associated with prescriptive inappropriateness identified only not participating in an ASP (*p* = 0.04) as the independent predictor of prescriptive inappropriateness (Table 3).

### 3.4. Factors Associated with Inappropriateness in Surgical Units

No factor was identified as associated with inappropriateness in antimicrobial prescriptions in surgical units, except being a unit not participating in an ASP: of the 88 antimicrobial prescriptions in non-ASP units, 75 (85.2%) were inappropriate, higher than that observed among the 16 antimicrobial prescriptions recorded in ASP units (50%, OR 5.77, 95% CI 1.84–18.1, *p* = 0.001) (Table 4).

### 3.5. Factors Associated with Inappropriateness in ICUs

In ICUs, no differences in inappropriate antimicrobial prescriptions were observed between prescriptions in units participating or not in an ASP (Table 5). Inappropriate antimicrobial prescriptions were more frequently identified in cases of empirical therapy (95.2% vs. 53.9%), a difference not significant to the statistical analysis (*p* = 0.07); however, inappropriate prescriptions were recorded in the case of sepsis or septic shock (9.6% vs. 46.1%, *p* = 0.01) or bloodstream infections (4.8% vs. 38.5%, *p* = 0.02) (Table 5). Multivariable analysis of the factors associated with prescriptive inappropriateness identified no independent predictor of prescriptive inappropriateness (Table 6).

## 4. Discussion

Antibiotic resistance is rising to dangerously high levels all over the world. Overuse and misuse of antibiotics contribute to the acquisition and spread of infections due to antibiotic-resistant bacteria. Thus, knowing the degree of inappropriateness in antimicrobial prescriptions and the factors associated with the different hospital areas may be useful in the design of strategies to reduce inappropriateness in antimicrobial prescriptions and the development of multidrug-resistant microorganisms.

In the present study, the level of inappropriate prescribing in the two hospitals evaluated in Southern Italy was high in all three areas (over 50%): medical, surgical and ICUs. The reasons for the inappropriate antimicrobial prescriptions, however, were similar in the three areas evaluated: antimicrobial unnecessary and antimicrobial not recommended were the most frequent reasons for inappropriateness. In the ICUs, the unnecessary use of antibiotics prevailed, probably because of the management of patients with more severe conditions, with symptoms similar to those of bacterial infections [17,18].

The highest rate (79.8%) of inappropriateness in antimicrobial prescriptions was observed in the surgical area, where the prescriptions of peri-operative prophylaxis obviously prevail. In fact, as already indicated in the literature, surgical prophylaxis often does not adhere to the guidelines, especially in terms of duration [19] or choice of the type of antibiotic [20]. In our study, 10% of antimicrobial prescriptions in the surgical area were excessive in duration, and in 25.3% of cases, it was not recommended. To increase the appropriateness of peri-operative prophylaxis, we revised the antimicrobial prophylaxis guidelines of our hospitals and promoted its application.

Considering the factors associated with inappropriateness in antimicrobial prescriptions, the main factor in medical and surgical units was the absence of an antimicrobial stewardship program, so it would be useful to implement stewardship programs in all units of both centres. Hospital ASPs have shown a positive impact on antimicrobial consumption, antimicrobial appropriateness and the incidence of bloodstream infections due to multidrug-resistant microorganisms, with reduced length of hospitalisation and without an increase in mortality [21,22,23,24,25]. Antimicrobial stewardship not only includes limiting inappropriate use but also optimising antimicrobial selection, dosing, route and duration of therapy to maximise the clinical cure. In the present paper, ASPs do not seem to be associated with antimicrobial appropriateness in ICUs, probably because the knowledge of antimicrobial therapy was greater in this setting due to the management of critical patients.

The study shows some limitations, the most important of which is represented by the small sample size: all the data analysed were collected during a single week in each hospital, so a limited number of prescriptions have been included. This did not allow us in some cases to stratify the data according to all potentially relevant characteristics of patients, such as the disease severity or the source of infection. Furthermore, the cross-sectional design and the absence of pre-intervention data limited the possibility of fully evaluating the impact of the ongoing antimicrobial stewardship program on the appropriateness of prescriptions. Finally, we should consider that the definition of appropriateness is not universal and can significantly vary according to individual ethical considerations or concerns regarding the working context, such as the availability of rapid microbiological tests or local epidemiology of healthcare-associated infections [8]. Indeed, despite several efforts to standardise the meaning of “appropriate” antimicrobial prescription [26], many different definitions can be found in the literature [27].

In conclusion, the present paper suggests that an ASP may be useful especially in medical and surgical units with the aim of reducing the use of broad-spectrum antibiotics, favoring appropriateness in antimicrobial prescription and encouraging adherence to the guidelines of antibiotic prophylaxis in the surgical field. However, in ICUs, specific programs able to limit empirical therapies and encourage the collection of microbiological samples may be useful to set up targeted therapies and to design antimicrobial protocols according to the clinical presentation of the patients and the local epidemiological situation in infections by multidrug-resistant microorganisms.

## Figures and Tables

**Table 1:** Reasons for inappropriate antimicrobial prescription according to medical, surgical and intensive care units.

	Medical Units (A)	Surgical Units (B)	Intensive Care Units (C)	p	OR (95% CI)	Chi-Square Value
N° (%) of Inappropriate Prescriptions/Antimicrobial Prescriptions	35/65 (53.8)	83/104 (79.8)	25/39 (64.1)	A vs. B = **0.0003** A vs. C = 0.30 B vs. C = 0.052	0.29 (0.15–0.58) 0.65 (0.29–1.48) 2.21 (0.98–4.98)	12.8 1.05 3.78
Antimicrobial Unnecessary N° (%)	6 (17.1)	19 (22.9)	12 (48)	A vs. b = 0.48 A vs. c = **0.010** B vs. c = **0.015**	0.69 (0.25–1.93) 0.22 (0.07–0.73) 0.32 (0.13–0.82)	0.48 6.6 5.92
Antimicrobial Inadequate N° (%)	2 (5.7)	7 (8.4)	1 (4)	A vs. b = 0.72 A vs. c = 1 B vs. c = 0.68	0.66 (0.13–3.34) 1.45 (0.12–16.9) 2.21 (0.26–18.9)	0.26 0.09 0.55
Antimicrobial Not Recommended N° (%)	8 (22.9)	21 (25.3)	6 (24)	A vs. b = 0.78 A vs. c = 0.92 B vs. c = 0.87	0.87 (0.44–2.22) 0.94 (0.28–3.15) 1.07 (0.38–3.04)	0.08 0.01 0.02
Excessive Duration N° (%)	3 (8.6)	8 (9.6)	1 (4)	A vs. b = 1 A vs. c = 0.63 B vs. c = 0.68	0.88 (0.22–3.53) 2.25 (0.22–22.9) 2.56 (0.30–21.5)	0.03 0.49 0.80
Inadequate Method of Administration N° (%)	5 (14.3)	3 (3.6)	0	A vs. b = **0.049**	4.44 (1.01–19.7)	4.43
2 Reasons for Inappropriate Antimicrobial Prescription N° (%)	9 (25.7)	23 (27.7)	5 (20)	A vs. b = 0.82 A vs. c = 0.60 B vs. c = 0.44	0.90 (0.37–2.21) 1.38 (0.40–4.78) 1.53 (0.51–4.57)	0.05 0.27 0.59

**Table 2:** Factors associated with inappropriateness in antimicrobial agents in medical units.

Variables	Appropriate Prescription	Inappropriate Prescription	*p* Value	OR (95% CI)	Chi-Square Value
**N°**	30	35			
Antibiotic therapy, N° (%)	30 (100)	31 (88.6)	0.12	NA	3.65
Prescriptions in ASP, Units N° (%)	14 (46.7)	3 (8.6)	**0.0006**	9.33 (2.34–37.2)	12.14
Prescriptions in non-ASP, Units, N° (%)	16 (53.3)	32 (91.4)	**0.0006**	0.11 (0.03–0.43)	12.14
Empiric therapy, N° (%)	14 (46.7)	25 (71.4)	0.04	0.35 (0.13–0.98)	4.13
**Acquisition of Infection**					
**N° of Infections**	30	31			
Community-acquired infection, N° (%)	17 (56.7)	18 (58.1)	0.67	0.94 (0.34–2.61)	0.18
Nosocomial infection, N° (%)	6 (20)	6 (19.4)	0.77	1.04 (0.29–3.68)	0.004
**Source of infection**					
Unknown source, N° (%)	5 (16.7)	11 (35.5)	0.25	0.36 (0.11–1–22)	2.79
Pneumonia, N° (%)	5 (16.7)	9 (29)	0.55	0.49 (0.14–1.68)	1.3
Endocarditis/Cardiovascular, N° (%)	5 (16.7)	0	**0.02**	**NA**	5.6
Genito-urinary, N° (%)	5 (16.7)	2 (6.5)	0.25	2.9 (0.52–16.3)	1.57
Other Sources, N° (%)	10 (33.3)	9 (29)	0.71	1.22 (0.13–2.31)	0.13
**Microbiology specimen (haemocultures)**					
**N° (%) Patients with Haemocultures**	25 (83.3)	18 (58.1)	0.048	3.6 (1.09–11.9)	4.68

**Table 3:** Logistic regression analysis for independent factors related to prescriptive inappropriateness in medical units.

Factor	OR	95% CI		p Value
**Lower Limit**			**Upper Limit**	
Specific vs. Empiric Antibiotic Therapy	0.60	0.11	3.10	0.54
Non-ASP Units vs. ASP Units	7.09	1.06	47.42	0.04
Haemocultures Yes vs. No	0.86	0.25	2.95	0.81
Source of Infection: Endocarditis vs. Not Endocarditis	0.08	0.01	1.07	0.053

**Table 4:** Factors associated with inappropriateness in antimicrobial agents in surgical units.

Variables	Appropriate Prescriptions	Inappropriate Prescriptions	*p* Value	OR (95% CI)	Chi-Square Value
**N°**	21	83			
Antibiotic Prophylaxis, N° (%)	15 (71.4)	54 (65.1)	0.58	1.34 (0.47–3.83)	0.30
Antibiotic Therapy, N° (%)	6 (28.6)	29 (34.9)	0.58	0.74 (0.26–1.13)	0.30
Prescriptions in ASP, Units N° (%)	8 (38.1)	8 (9.7)	**0.001**	5.77 (1.84–18.1)	12.5
Prescriptions in Non-ASP, Units, N° (%)	13 (61.9)	75 (90.4)	**0.001**	0.17 (0.06–0.54)	12.5
Empiric Therapy, N° (%)	4 (66.7)	27 (93)	0.29	0.49 (0.15–1,59)	1.46
**Acquisition of infection**					
**N° of Infections**	6	29			
Community-Acquired Infection, N° (%)	3 (50)	14 (48,3)	1	1.07 (0.18–6.22)	0.006
Nosocomial Infection, N° (%)	2 (33.3)	8 (27.6)	1	1,21 (0.20–8.62)	0.08
**Severity of infection**					
No Sepsis, N° (%)	5 (83.3)	22 (75.9)	1	1.59 (0.16–16.0)	0.16
**Microbiology specimen**					
**N° of collections**	2 (33.3)	8 (27.6)	1	1.31 (0.20–8.62)	0.08

**Table 5:** Factors associated with inappropriateness in antimicrobial agents in ICUs.

Variables	Appropriate Prescriptions	Inappropriate Prescriptions	*p* Value	OR (95% CI)	Chi Square Value
**N°**	14	25			
Antibiotic Therapy, N° (%)	13 (92.9)	21 (84)	0.64	2.47 (0.25–24.6)	0.63
Prescriptions in ASP, Units N° (%)	5 (35.7)	3 (12)	0.11	4.07 (0.80–20.7)	3.09
Prescriptions in Non-ASP, Units, N° (%)	9 (64.3)	22 (88)	0.11	0.24 (0.05–1.25)	3.09
Empiric Therapy, N° (%)	7 (53.9)	20 (95.2)	0.07	0.25 (0.06–1.25)	3.8
**Acquisition of infection**					
**N° of infections**	13	21			
Community-Acquired Infection, N° (%)	10 (76.9)	12 (57.1)	0.29	2.5 (0.53–11.8)	1.38
Nosocomial Infection, N° (%)	3 (23.1)	7 (33.3)	0.70	0.60 /0.12–2.90)	0.41
**Severity of infection**					
Sepsis or Septic Shock, N° (%)	6 (46.1)	2 (9.6)	**0.01**	8.14 (6.32–9.96)	5.98
No Sepsis, N° (%)	6 (46.2)	14 (66.7)	0.24	0.43 (0.10–1.77)	1.39
**Source of infection**					
Pneumonia, N° (%)	5 (38.5)	7 (33.3)	1	1.25 (0.30–1.27)	0.09
Abdominal, N° (%)	1 (7.7)	5 (23.8)	0.37	0.27 (0.03–2.59)	1.43
Bloodstream, N° (%)	5 (38.5)	1 (4.8)	**0.02**	12.5 (1.26–124.5)	6.27
Other sources, N° (%)	2 (15.4)	8 (38.1)	0.16	0.3 (0.05–1.69)	1.99
**Microbiology Specimen**					
**N° of Collections**	10 (76.9)	10 (47.6)	0.15	3.67 (0.78–17.2)	2.85

**Table 6:** Logistic regression analysis for independent factors related to prescriptive inappropriateness in ICUs.

Factor	OR	95% CI		*p* Value
Lower Limit			Upper Limit	
Empiric vs. Specific Antibiotic Therapy	0.44	0.23	2.91	0.41
Severity of Infection: No Sepsis vs. Sepsis or Septic Shock	7.00	060	81.68	0.12
Source of Infection: Bloodstream vs. Not Bloodstream Infection	0.31	0.11	6.17	0.83

## Data Availability

The datasets used and/or analysed during the current study are available from the corresponding author on reasonable request.

## Supplementary Materials

The following are available online at https://www.mdpi.com/article/10.3390/life11060475/s1, Table S1: The units evaluated in Naples, Table S2: The units evaluated in Caserta, Table S3: Characteristics of units and antimicrobial prescription according to medical, surgical and intensive care units, Table S4: Prescription of the different antimicrobials according to medical, surgical and intensive care units.
