# Supplementary material for: Inappropriateness of Antibiotic Prescribing in Medical, Surgical and Intensive Care Units: Results of a Multicentre Observational Study
Source: Life (Basel). 2021 May 24;11(6):475. doi: 10.3390/life11060475 (PMC8225033; doi:10.3390/life11060475)
Supplement: Supplementary file 1 [file life-11-00475-s001.zip › life-1204119-supplementary.pdf]

life

Supplementary Materials

Inappropriateness of Antibiotic Prescribing in Medical, Surgical and Intensive Care Units: Results of a Multicentre Observational Study

Table S1. The Units evaluated in Naples.

Napoli Units		
**Medical Units (10)**	**Surgical Units (16)**	**Intensive Care Units (3)**
Neurology	Oncological Surgery of Digestive system	Intensive Care Unit
Gastroenterology	4th General Surgery	Neonatal Intensive Care Unit
Rheumatology	Thyroid Surgery	Intensive Care Unit number 2
Infectious Diseases	General and Geriatric Surgery	
Nephrology	9th General Surgery	
Psychiatry and Involuntary Psychiatric Treatment	Plastic Surgery	
Internal Medicine	Orthopedics	
Geriatrics	Maxillo-Facial Surgery and Pedodontics	
Endocrinology	Pediatric Surgery	
Pediatrics and pediatric onco-hematology	Ginecology and Obstetrics	
	Otolaryngology	
	Urology	
	Thoracic Surgery	
	7th General Surgery	
	Digestive System Surgery	
	11th General Surgery	

Table S2. The Units evaluated in Caserta.

Caserta Units		
**Medical Units (9)**	**Surgical Units (9)**	**Intensive Care Units (6)**
Geriatrics	Orthopedics	Emergency Surgery and Trauma Centre
Pediatrics	Short hospitalization Surgery	Neonatal Intensive Care Unit
Onco-Hematology	Urology	Coronary Intensive Care Unit
Infectious Diseases	General and Oncological Surgery	Emergency Medicine
Pneumology	Otolaryngology	Intensive Care Unit after Heart Surgery
Internal Medicine	Maxillo-Facial Surgery	Anesthesia
Nephrology and Dialysis	Ginecology and Obstetrics	
Gastroenterology	Vascular Surgery	
Neurology	Heart Surgery	

Table S3: Characteristics of units and antimicrobial prescription according to medical, surgical and intensive care units.

Variables	Medical Units	Surgical Units	Intensive Care Units
		Characteristics of Units	
Units, N° (%)	19 (35.9)	25 (47.2)	9 (16.9)
Beds available, N°	232	269	109
Patients assessed/Occupancy rate beds, N° (%)	210 (90.5)	187(69.5)	89 (81.7)
Units in ASP, N° (%)	5 (26.3)	6 (24)	4 (44.4)
		Characteristics of antimicrobial prescriptions	
**N° of Antimicrobial prescriptions**	65 (31%)	104 (55,6%)	39 (66,3%)
Antibiotic prophylaxis N° (%)	4 (6.2)	69 (66.3)	5 (12.8)
Antimicrobial therapy N° (%)	61 (93.8)	35 (33.7)	34 (87.2)
		Prescriptive appropriateness	
**N° of Antimicrobial prescriptions**	65	104	39
Inappropriate antimicrobial prescription N° (%)	35 (53.8)[A]	83 (79.8)[B]	25 (64.1)[C]

Notes: A vs. B, p= **0.0003;** A vs. C, p= 0.30; B vs. C, p = 0.052

Table S4. Prescription of the different antimicrobials according to medical, surgical and intensive care units.

Variables	Medical Units	Surgical Units	Intensive Care Units	P value
		Antibiotics		
**N° of Antibiotics**	91	121	60	
Aminopenicillin, N° (%)	12 (13.2)	37 (30.6)	8 (13.3)	A vs b=**0.0029**<br>A vs c=0.98<br>B vs c=**0.011**
Piperacillin/tazobactam, N° (%)	13 (14.3)	7 (5.8)	11 (18.3)	A vs b=**0.036**<br>A vs c=0.50<br>B vs c=**0.008**
Carbapenem, N° (%)	2 (2.2)	4 (3.3)	7 (11.7)	A vs b=0.70<br>A vs c=**0.03**<br>B vs c=**0.043**
First-generation cephalosporins, N° (%)	0	28 (23.2)	2 (3.3)	A vs b=**0.00001**<br>A vs c=0.16<br>B vs c=**0.0005**
Third-generation cephalosporins, N° (%)	20 (22)	13 (10.7)	5 (8.3)	A vs b=**0.025**<br>A vs c=**0.027**<br>B vs c=0.61
Macrolides, N° (%)	4 (4.4)	1 (0.8)	2 (3.3)	A vs b=0.17<br>A vs c=1<br>B vs c=0.25
Lincosamides, N° (%)	0	2 (1.7)	0	A vs b=0.51<br>A vs c=1<br>B vs c=1
Aminoglycosides, N° (%)	2 (2.2)	1 (0.8)	6 (10)	A vs b=0.58<br>A vs c=**0.02**<br>B vs c=**0.006**
Quinolones, N° (%)	13 (14.3)	13 (10.7)	4 (6.7)	A vs b=0.44<br>A vs c=0.19<br>B vs c=0.43
Glycopeptides, N° (%)	8 (8.8)	3 (2.5)	2 (3.3)	A vs b=**0.06**<br>A vs c=0.32<br>B vs c=1

Oxazolidinone, N° (%)	0	1 (0.8)	3 (5)	A vs b=1<br>A vs c=**0.06**<br>B vs c=0.11
Cotrimoxazole, N° (%)	4 (4.4)	2 (1.7)	0	A vs b=0.76<br>A vs c=0.15<br>B vs c=1
Metronidazole, N° (%)	4 (4.4)	7 (5.8)	4 (6.7)	A vs b=0.76<br>A vs c=0.71<br>B vs c=0.75
Colistin, N° (%)	0	0	2 (3.3)	A vs b=1<br>A vs c=0.16<br>B vs c=0.11
Lipopetide, N° (%)	1 (1.1)	0	0	A vs b=0.43<br>A vs c=1<br>B vs c=1
Tygecicline, N° (%)	0	1 (0.8)	0	A vs b=1<br>A vs c=1<br>B vs c=1
Azole, N° (%)	2 (2.2)	1 (0.8)	3 (5)	A vs b=0.58<br>A vs c=0.39<br>B vs c=0.11
Echinocandin/Amphotericin B, N° (%)	2 (2.2)	0	1 (1.7)	A vs b=0.18<br>A vs c=1<br>B vs c=0.33
Antitubercolosis Agents, N° (%)	4 (4.4)	0	0	A vs b=**0.03**<br>A vs c=0.15<br>B vs c=1
	Way of administration			
**N° of Antibiotics**	91	121	60	
Oral, N° (%)	19 (21.4)	15 (12.5)	4 (6.3)	A vs b=0.096<br>A vs c=**0.02**<br>B vs c=0.31
Intravenous, N° (%)	72 (80.9)	106 (88.3)	56 (87.5)	A vs b=0.096<br>A vs c=**0.02**<br>B vs c=0.31
